# Single-photon superabsorption in CsPbBr_3_ perovskite quantum dots

**DOI:** 10.1038/s41566-025-01684-3

**Published:** 2025-05-21

**Authors:** Simon C. Boehme, Tan P. T. Nguyen, Chenglian Zhu, Ihor Cherniukh, Leon G. Feld, Dmitry N. Dirin, Maryna I. Bodnarchuk, Claudine Katan, Jacky Even, Maksym V. Kovalenko, Gabriele Rainò

**Affiliations:** 1 Institute of Inorganic Chemistry, Department of Chemistry and Applied Biosciences, ETH Zürich, Zürich, Switzerland; 2 Laboratory for Thin Films and Photovoltaics, Empa—Swiss Federal Laboratories for Materials Science and Technology, Dübendorf, Switzerland; 3 Univ. Rennes, ENSCR, CNRS, ISCR (Institut des Sciences Chimiques de Rennes)—UMR6226, Rennes, France; 4 Univ. Rennes, INSA Rennes, CNRS, Institut FOTON—UMR6082, Rennes, France

**Keywords:** Quantum dots, Quantum dots, Quantum dots

## Abstract

The absorption of light via interband optical transitions plays a key role in nature and applied technology, enabling efficient photosynthesis and photovoltaic cells, fast photodetectors or sensitive (quantum) light–matter interfaces. In many such photonic systems, enhancing the light absorption strength would be beneficial for yielding higher device efficiency and enhanced speed or sensitivity. So far, however, cavity-free light absorbers feature poorly engineerable absorption rates, consistent with the notion that the coupling strength between the initial and final states is an intrinsic material parameter. By contrast, greatly enhanced absorption rates had been theoretically predicted for superradiant systems, which feature giant oscillator strength through spatially extended coherent oscillations of the electron polarization. Unlike for emission processes, however, experimental realizations of superradiance in absorption—‘superabsorption’—remain sparse and require complicated excited-state engineering approaches. Here we report superabsorption by the time reversal of single-photon superradiance in large CsPbBr_3_ perovskite quantum dots. Optical spectroscopy reveals a bandgap absorption that strongly increases with the quantum dot volume, consistent with a giant exciton wavefunction. Configuration-interaction calculations, quantitatively agreeing with the experiment, attribute the observed single-photon superabsorption to strong electron–hole pair-state correlations. The approach brings new opportunities for the development of more efficient optoelectronic devices and quantum light–matter interfaces.

## Abstract (web summary)

Greatly enhanced light absorption is reported in large perovskite quantum dots by realizing a transition with a giant oscillator strength at the optical bandgap.

## Main

Optical transitions with high oscillator strength are key resources for many optical, electro-optical, biophotonic and quantum information processing applications that use semiconductors and their nanostructures as the active medium. Light-harvesting and light-emitting devices, such as solar photovoltaic cells, lasers and light-emitting diodes, profit from a large absorption cross-section. Unfortunately, increasing the absorption cross-section is challenging, as it represents an intrinsic material property set by the specific energy levels involved in the transition.

**Fig. 1:**
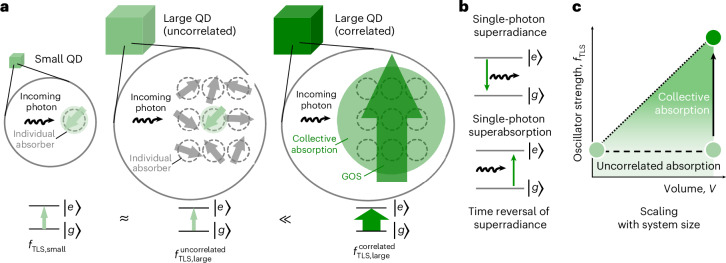
Single-photon superabsorption in solid-state emitters as the time reversal of single-photon superradiance. **a**, Light absorption at the bandgap of semiconductor QDs can be likened to the excitation of a two-level system; in a simplified view, one may picture a large QD (middle schematic) as a multiple of a small QD (left schematic); the oscillator strength of a conventional two-level system is size independent, that is, ${f}_{{{\rm{TLS}}},{{\rm{small}}}}^{\;{{\rm{uncorrelated}}}} \approx {f}_{{{\rm{TLS}}},{{\rm{large}}}}^{\;{{\rm{uncorrelated}}}}$; in a superabsorbing two-level system, however, correlations among the individual absorption contributions enable a giant collective dipole (right schematic) with enhanced system oscillator strength ${f}_{{{\rm{TLS}}},{{\rm{large}}}}^{\;{{\rm{correlated}}}}\gg {f}_{{{\rm{TLS}}},{{\rm{large}}}}^{\;{{\rm{uncorrelated}}}}$. **b**, Single-photon superabsorption may be realized by the time reversal of single-photon superradiance. **c**, Oscillator strength *f*_TLS_ of the two-level system is constant for an uncorrelated electron–hole pair (horizontal dashed line) but increases with the system volume for a superabsorbing (highly correlated) electron–hole pair (dotted arrows).

The advent of nanotechnology had initially raised hopes that reduced material dimensionality may represent a lever to enhance light absorption. For example, early theoretical work predicted that compared with their bulk counterparts, epitaxial III–V nanostructures may display enhanced material gain^1^, a quantity directly related to the absorption cross-section via the Einstein coefficients. Furthermore, electronic correlations in nanostructures may increase the oscillator strength, since the latter is proportional to the probability of finding the electron and hole at the same position^2–5^. Interestingly, however, experimental verifications of such early theoretical predictions are still lacking. For example, a quantitative experimental work on epitaxial III–V quantum dots (QDs) and quantum wells inferred that the absorption strength scales linearly with the volume of the structure, regardless of the used quantum confinement^6^. This experience of size-independent intrinsic (that is, per-unit-volume) absorption coefficients energetically well above the bandgap is generally accepted and by now routinely being used to determine QD concentrations from linear absorption measurements at higher energies, typically in the (near-)ultraviolet spectral region^7,8^. At the bandgap, the photophysics is generally more complex and less explored. Recently, it has been suggested that in the absence of excitonic effects, the absorption strength follows a universal scaling law: all direct bandgap semiconductors, regardless of their composition and dimension, display a band-edge absorptance of π*α* per exciton diameter, where *α* is the fine-structure constant^9,10^. This is consistent with the notion that an idealized semiconductor nanostructure may be regarded as a two-level system in the electron–hole picture with size-independent oscillator strength (Fig. 1). With few experimental exceptions^7^, such an analogy is considered valid for a QD in the strong-confinement regime in the absence of electronic correlations and considering the first two electron–hole pair states^11^.

The current state of the art on light absorption by colloidal and epitaxial nanostructures suggests two conclusions. First, the effect of quantum confinement on the absorption process remains poorly understood, presumably due to a complex interplay of quantum confinement, valence-band degeneracy, and inhomogeneous broadening related to size and shape distribution^7^. Second, the light absorption strength of semiconductors seemingly remains immune to experimental engineering approaches via material dimensionality and composition.

In contrast to light absorption rates, the radiative rate of band-edge emissive states has been the target of many successful engineering efforts. In general, spontaneous photon emission in semiconductors results from the spontaneous radiative recombination of excitons induced by zero-point fluctuations of the vacuum mode of the electromagnetic field. This leads to an excited state with a finite lifetime, described by Fermi’s golden rule as

1 $${{\varGamma }}_{e\to g}=\frac{2\uppi }{\hslash }{|\langle g|H^{\prime} |e\rangle |}^{2}\rho ({E}_{{\rm{f}}}),$$

where the transition rate *Γ*_*e*→*g*_ from the excited state |*e*〉 to the ground state |*g*〉 is proportional to the product of the modulus squared of the matrix element describing the optical transition |〈*g*|*H*′|*e*〉|^2^ and the local density of photonic states *ρ*(*E*_f_) (LDOS) at the emitting energy *E*_f_. The quest for enhanced radiative decay rates has mostly focused on exploiting plasmonic or dielectric microcavities^12,13^, thereby enhancing the LDOS by the so-called Purcell effect. However, accelerated spontaneous radiative decay may also be achieved in a cavity-free system, as suggested by Robert Dicke in 1954. In his ground-breaking work on superradiance^14^, a collection of dipoles confined to a volume much smaller than the wavelength of the light field could radiate much faster than individual (uncoupled) dipoles on their own. At the heart of this many-body process are correlations between dipoles that self-align to form a giant dipole moment with an enhanced radiative rate proportional to *N*^2^, where *N* is the number of correlated dipoles. Such a burst of photons has also been termed superfluorescence^15^. This process has been widely explored^16–25^ theoretically and experimentally and proliferated the development of novel concepts including the superradiant laser^26^ and efficient photon-retrieval schemes for quantum communication protocols^27,28^.

A tempting question is whether the process of superradiance/superfluorescence and its exciting features can be time reversed. Time-reversal symmetry in quantum mechanics would imply that systems with enhanced emission rates also feature enhanced absorption rates. Although there are no fundamental limitations to time reversing the superradiance process, its practical implementation is not straightforward. If coupling and synchronization of the *N* individual dipoles only occur after photoexcitation, as in the scheme originally considered by Dicke, superabsorption will require complex pumping schemes for the preparation of the giant dipole. Accordingly, this process has only recently been demonstrated and, so far, only for an atomic system^29^, whereas remaining elusive in commercially relevant systems such as semiconductor nanostructures.

By contrast, we anticipate that an alternative realization of superabsorption could be achieved by time reversing single-photon superradiance^30,31^. The latter process, involving a single quantum of energy distributed coherently over the entire quantum emitter volume, has so far been demonstrated in the spontaneous emission of epitaxial QDs^30^, colloidal perovskite QDs^31^ and glass-embedded CuCl QDs^32^. Such single-photon superradiance bears analogies and differences to the canonical Dicke superradiance. A strong formal analogy exists in the nature of the emitting state: in its Wannier-site-localized form, the exciton wavefunction of the single-photon emitter represents a product state with a correlation term arising from the *N* contributing unit cells, as typical for a Dicke superradiant state^31^. However, single-photon superradiance also differs from the canonical Dicke superradiance in several important aspects. First, the acceleration of the spontaneous emission process does not arise from the cooperativity of *N* excitons (as in superfluorescence), but from strong intra-exciton correlations^33–35^, which induce a very high oscillator strength. Second, the radiative rate does not display the characteristic superlinear scaling with the excitation intensity but scales with the number of coherently coupled unit cells, experimentally observed via an increase in the bandgap transition strength in larger QDs (Fig. 1a). Third, single-photon superradiance is at play immediately on photoexcitation, that is, without the need for a synchronization build-up. In particular, the latter aspect greatly facilitates the experimental realizations of superabsorption: although proposals relying on time reversing the canonical Dicke superradiance were explicitly concerned with the question how to excite and keep the system in an excitation state close to the middle of the Dicke ladder^29,36,37^, this challenging requirement is circumvented when time reversing the single-photon superradiance process (Fig. 1b). The latter could be conveniently realized with simple excitation protocols, such as readily available weak and incoherent continuous-wave light sources and directly probed by linear absorption measurements (Fig. 1c).

As a versatile and commercially relevant material platform for demonstrating single-photon superabsorption, we choose colloidal CsPbX_3_ (X = Cl, Br or I) lead halide perovskite QDs, which have attracted immense research efforts since their first hot-injection synthesis in 2015 (ref. ^38^). Their appeal lies in the facile yet precise control over the QD size, shape and composition, as well as superior optical properties such as spectrally narrow photoluminescence (PL), high brightness and near-unity quantum yield facilitated by a defect-tolerant electronic structure^39–41^. Such unique features, achieved with simple^42^ and versatile^43^ room-temperature solution-chemistry methods, amenable for scale-up and industrial processing, have already yielded perovskite-QD-based optoelectronic devices with unprecedented performance, including light-emitting diodes with external quantum efficiencies above 25% (ref. ^44^), lasers with wide spectral tunability^45^ and photodetectors with high responsivity^46^. Parallel to the development of classical light-emitting devices, CsPbX_3_ QDs are also explored as sources of quantum light, capable of either delivering single photons^47,48^ or bunched (temporally correlated) multiphoton bundles^22^ when organized in QD superlattices^49^.

Here we leverage the mature synthetic chemistry protocols for perovskite QDs to demonstrate single-photon superabsorption via the observation of a giant-oscillator-strength (GOS)^50^ transition in large CsPbBr_3_ perovskite QDs. The band-edge absorption exhibits a strong increase with particle volume, with large and weakly confined QDs displaying almost an order of magnitude larger optical transition strength compared with small and strongly confined QDs. Theoretical calculations based on the configuration-interaction formalism corroborate the experimental results and attribute the band-edge GOS to strong electron–hole pair-state correlations and a large delocalization of the exciton wavefunction^31^.

## Results

### Broad size tunability in colloidal CsPbBr_3_ perovskite QDs

**Fig. 2:**
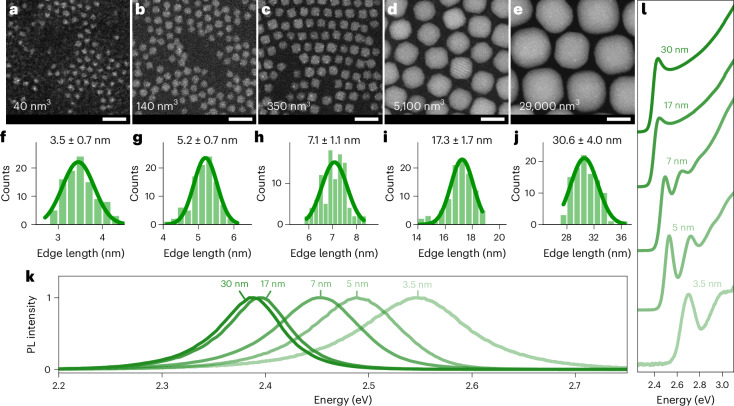
CsPbBr_3_ QDs spanning three orders of magnitude in volume. **a**–**e**, Scanning transmission electron microscopy images of QDs with mean edge lengths of about 3.5 nm (**a**), 5 nm (**b**), 7 nm (**c**), 17 nm (**d**) and 30 nm (**e**), and the corresponding volumes of about 40 nm^3^, 140 nm^3^, 350 nm^3^, 5,100 nm^3^ and 29,000 nm^3^, respectively. Scale bars, 20 nm. **f**–**j**, Corresponding histograms of the QD mean edge length in **a**–**e** (**f**–**j**, respectively). **k**, PL spectra of the QD colloids at room temperature. **l**, Normalized absorbance spectra of the QD colloids at room temperature; for clarity, subsequent spectra are shifted vertically.

We have synthesized a series of colloidal QDs spanning about three orders of magnitude in volume, that is, with edge lengths ranging from 3.5 nm to 30 nm, extending from the strong-confinement to weak-confinement regime. Representative transmission electron microscopy images (Fig. 2a–e) demonstrate a high size and shape uniformity (with a standard deviation of the QD size on the order of 10%; Fig. 2f–j), which translates into a small inhomogeneous line broadening, manifested in the narrow-band PL spectra (Fig. 2k) and well-resolved absorption features (Fig. 2l). All the samples exhibit an orthorhombic crystal structure from cryogenic temperature up to room temperature. Detailed information on the sample preparation and the used optical spectroscopy setup are reported in the Methods. In the following, we will probe whether this system exhibits the hallmarks of single-photon superabsorption such as a bandgap absorption strength, which increases with increasing QD size (Fig. 1c).

### GOS of the 1S transition

**Fig. 3:**
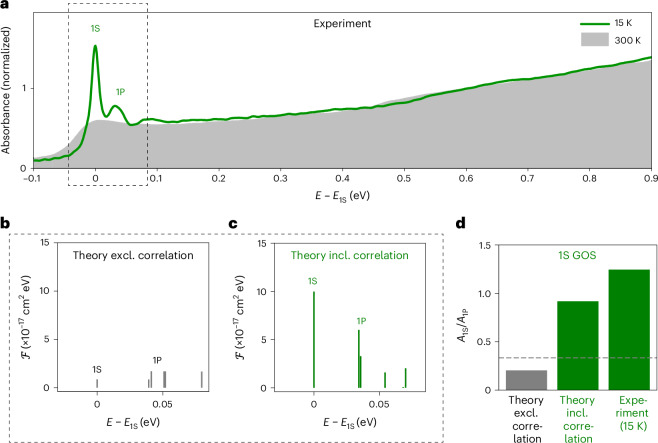
GOS at the bandgap (1S) transition in 17-nm CsPbBr_3_ QDs enabled by strong electronic correlations at cryogenic temperature. **a**, Experimental absorption spectrum at 15 K (line) and 300 K (shaded area); cooling to 15 K greatly enhances the 1S absorption strength, weakly enhances the 1P transition and negligibly affects the higher-energy transitions. **b**,**c**, Theoretical one-photon transition strength ${\mathcal{F}}$ for the lowest-energy states excluding (**b**) and including (**c**) correlations, as defined in Supplementary Equation (15); the energy scale (*E* – *E*_1S_) is referenced to the bandgap *E*_1S_. **d**, Ratio *A*_1S_/*A*_1P_ of the absorbance at the 1S and 1P state; the experimental ratio at 15 K (right bar) is quantitatively reproduced by the CI-EMA-based theory when accounting for electron–hole pair-state correlations (middle bar) but not when neglecting it (left bar). The dashed horizontal line indicates a ratio of 1/3 that would be anticipated solely based on the degeneracy of the 1S and 1P transitions.

Figure 3a illustrates the measured linear absorption spectra recorded at room temperature and cryogenic temperature (15 K) of a thin film of 17-nm CsPbBr_3_ QDs. At room temperature, only a single absorption peak near the bandgap (1S transition, at about 2.4 eV) is discernable, superimposed over a broad continuum-like density of states. The latter is a common feature for such relatively large and weakly confined QDs since the higher-energy transitions (1P, 1D and so on) are separated by less than their respective homogeneous line broadening at room temperature. Remarkably, cooling to 15 K dramatically alters the absorption spectrum. First, the series of 1S, 1P, 1D and higher-order transitions becomes well resolved due to the now-negligible homogeneous line broadening after freezing out most crystal vibrations. Second, and more importantly, cooling enhances the optical absorbance at the lowest-energy (1S) transition, suggesting an increase in the 1S oscillator strength at cryogenic temperature. The absorption cross-section of this transition is typically three times weaker than that of the second (1P) transition, due to the threefold degeneracy of the latter. Therefore, our observation of a much stronger 1S transition compared with the 1P transitions, but consistent with recent all-order correlation calculations^4^, is surprising and deserves a detailed analysis for a comprehensive rationalization.

To this end, we performed theoretical calculations using the configuration-interaction method based on the effective-mass approximation (CI-EMA) and considering parameters relevant to the orthorhombic crystal structure at low temperature, with a reduced effective mass *μ* = 0.126, and an effective dielectric constant *ε*_eff_ = 7.3 (Supplementary Note 1). The CI-EMA method captures electronic correlation effects in both single- and multiexciton regimes, as confirmed by the well-reproduced experimental binding energies of exciton complexes (for example, trions and biexcitons) obtained by single-QD spectroscopy^51,52^. To probe the effect of electronic correlation in the single-exciton regime studied here, we calculate the one-photon transition strength spectrum for a weakly confined CsPbBr_3_ QD as electronic correlations are either neglected or included, respectively. In the absence of any correlation (Fig. 3b), the 1S transition strength is equal to or weaker than those of an average 1P transition (Supplementary Note 1), 1D transition and higher-energy transitions; such a spectrum resembles the experimentally obtained absorption spectrum at room temperature, when considering the experimental line broadening. Including electronic correlations drastically enhances the 1S transition strength by about an order of magnitude, slightly enhances the 1P transitions and hardly enhances the higher-energy transitions (Fig. 3c and Supplementary Fig. 2); such a spectrum resembles the experimental low-temperature absorption spectrum. Overall, the remarkable agreement between the experimental low-temperature absorption spectrum and CI-EMA calculation including electron–hole pair-state correlations and the agreement between experimental room-temperature absorption spectrum and CI-EMA calculation neglecting correlations suggests that (1) the developed CI-EMA model captures the salient features of the optical response of CsPbBr_3_ QDs; (2) electronic correlations are at the origin of the giant 1S absorption strength at cryogenic temperature; and (3) the giant 1S absorption strength and correlation effects are lost by heating to room temperature, probably due to a strong exciton–phonon coupling (Supplementary Note 1 and Supplementary Fig. 1).

In the following, we aim at quantifying the 1S absorption enhancement. Although we provide the absolute enhancement of both 1S and 1P transitions in our theory section (Supplementary Fig. 2), such an absolute quantification is more challenging in the experiment, due to sizable error margins in experimentally determining the volume filling fraction of QDs in the thin films and, consequently, the absolute per-unit-volume absorbances for all the studied QD thin films. Further uncertainties arise from dielectric screening effects. Experimentally, we, therefore, aim for a more robust relative enhancement metric, which is intrinsically independent of the films’ QD filling fraction and thickness and thereby yields a more accurate QD size dependence. A detailed motivation and explanation for our used experimental method is provided in the Methods and Supplementary Note 3. Briefly, we opt to reference the 1S absorbance to the absorbance at a higher-energy transition at which the correlation-induced enhancement is weaker. Specifically, to account for spectrally overlapping absorbance features, especially for ensembles of large QDs, we report the 1S–1P absorbance ratio *A*_1S_/*A*_1P_, derived from multipeak spectral fitting (Supplementary Fig. 3). This experimentally accessible ratio, when multiplied by the degeneracy ratio, represents a conservative estimate for the absolute magnitude of the 1S enhancement. Without enhancement, we expect roughly equal absorption cross-sections in the 1S transition and each of the three 1P transitions (that is, 1P_*x*_, 1P_*y*_ and 1P_*z*_), yielding *A*_1S_/*A*_1P_ ≈ 1/3. Figure 3d shows that a similar ratio is indeed found theoretically when neglecting correlation. By contrast, including electronic correlation in the CI-EMA calculation yields a markedly increased *A*_1S_/*A*_1P_ ≈ 1, that is, an enhancement of about a factor of 3, in agreement with the low-temperature experiment.

### Volume scaling

**Fig. 4:**
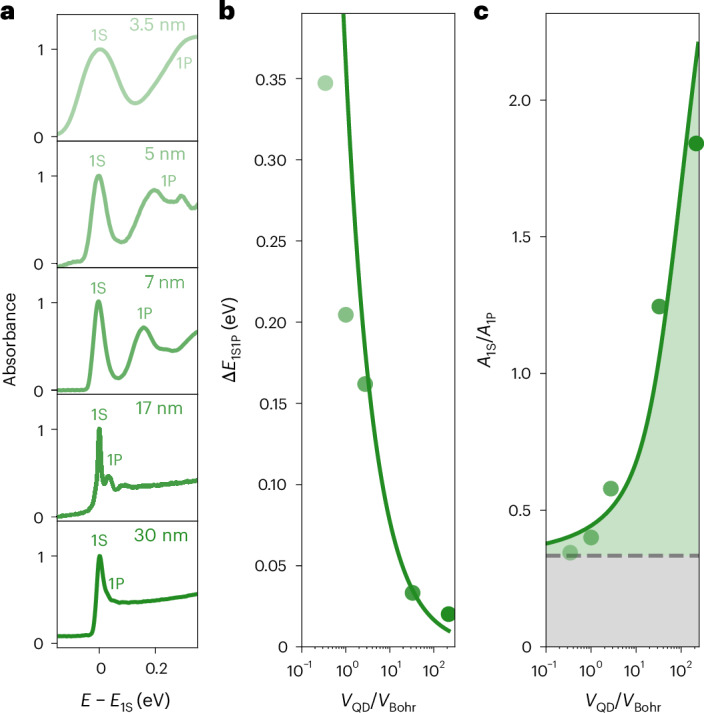
Size-dependent single-photon superabsorption in CsPbBr_3_ QD thin films. **a**, Experimental absorbance spectra of QD thin films at 15 K for mean QD sizes of 3.5 nm, 5 nm, 7 nm, 17 nm and 30 nm; the energy scale (*E* – *E*_1S_) is referenced to the respective bandgap *E*_1S_, the absorbance is normalized to the 1S peak, and the 1S and 1P peaks are indicated with labels. **b**, Experimental size-dependent energy separation Δ*E*_1S1P_ between the 1S and 1P transition (markers) coarsely follows the trend based on a simple particle-in-a-box model (solid line); the QD size is expressed in multiples of the Bohr cube volume (*V*_QD_/*V*_Bohr_), assuming a Bohr radius of 3.1 nm. **c**, With increasing QD size, the 1S–1P absorbance ratio *A*_1S_/*A*_1P_ at 15 K (markers) increases by about one order of magnitude; QDs lacking electronic correlation would exhibit a much weaker and size-independent *A*_1S_/*A*_1P_ ratio close to the associated degeneracy ratio, that is, 1/3 (dashed horizontal line); after including electronic correlation effects, CI-EMA calculations (solid line) quantitatively reproduce the experimental trend.

To unequivocally assign the experimentally and theoretically found 1S GOS to superabsorption, we check for another hallmark of this process, that is, a scaling of the absorbance with system size (Fig. 1c)^2,29,36^. Figure 4a reports the experimental 15-K absorbance spectra for QDs with edge lengths increasing from 3.5 nm up to 30 nm. For QDs in the strong-confinement regime, that is, for QDs smaller than the Bohr diameter of 6–7 nm in CsPbBr_3_ (ref. ^38^), the absorption spectrum features two broad yet well-separated peaks, assigned to the 1S and 1P transitions. Both large (mainly inhomogeneous) broadening and large 1S–1P energy separation for such small QDs result from the well-known quantum confinement effect, with 1S–1P splitting energies in good agreement with a simple particle-in-a-box model scaling with the QD volume as ${V}_{{{\rm{QD}}}}^{-2/3}$ (Fig. 4b).

The marked size-dependent increase in the *A*_1S_/*A*_1P_ absorbance ratio is, however, unique (Fig. 4c). For the smallest and strongly confined 3.5-nm QDs, the 1S optical transition features an absorbance 0.34 times the one at the 1P optical transition, very close to the degeneracy ratio of both peaks (1/3), indicating an equal oscillator strength in the 1S transition and the average of the three 1P transitions. When progressively increasing the QD size to 30 nm, however, *A*_1S_/*A*_1P_ increases by more than a factor 5, yielding a 1S transition almost twice as absorbing as the three 1P transitions combined. As mentioned, this size-dependent increase in *A*_1S_/*A*_1P_ represents only a conservative estimate for the absolute correlation-induced 1S absorbance enhancement. Accounting for some enhancement also in the 1P transitions, the absolute experimental 1S enhancement from single-photon superabsorption may even exceed one order of magnitude. Indeed, our CI-EMA calculations reveal a correlation-induced absolute 1S enhancement approaching a factor of 12 already for the second-largest QD size of 17 nm (Supplementary Fig. 2b). A similar conclusion can be inferred from the experimental data (Supplementary Fig. 4). Importantly, the CI-EMA calculations also reproduce—even quantitatively—the experimental size dependence in Fig. 4c, thereby reaffirming the critical role played by electronic correlations and the occurrence of single-photon superabsorption in large CsPbBr_3_ QDs with excitons in the weak-confinement regime.

## Discussion

Our study experimentally reports and mechanistically explains the first observation of superabsorption in a semiconductor nanostructure. In particular, such large CsPbBr_3_ QDs were recently shown to sustain single-photon superradiance^31^, with radiative rates as high as (100 ps)^−1^ at cryogenic temperature (Supplementary Figs. 5 and 6), more than an order of magnitude higher than the expected rate of ~(2.5 ns)^−1^ in the strong-confinement regime^4^. We suggest that both observations, that is, about an order of magnitude larger 1S absorbance and about an order of magnitude faster radiative decay from the 1S state, are intricately related (Supplementary Fig. 4), with the single-photon superabsorption reported here representing the time-reversal process of single-photon superradiance (Fig. 1b).

We further suggest that the occurrence of single-photon superabsorption in CsPbBr_3_ perovskite QDs at cryogenic temperature is enabled by the following fortunate set of enabling attributes. (1) The synthetic access to size- and shape-pure QDs^42^ with a volume more than two orders of magnitude larger than the Bohr volume. (2) The strong effect of correlations in this material afforded by the comparably pronounced excitonic system, with the bulk CsPbBr_3_ exciton binding energy of about 30 meV exceeding that of many prototypical semiconductors (for example, about 4 meV in bulk GaAs)^53^. (3) The low structural and electronic disorder at cryogenic temperature. (4) The simplicity of the electronic band structure, which, unlike for previously explored semiconductor nanostructures, closely approaches the simplistic picture of a collection of ‘two-level systems’ at the heart of Dicke’s prediction. The latter aspect is realized with a bandgap formed by two mirror-like parabolas, that is, with a curvature of the same magnitude but opposite sign, well separated from other valence and conduction bands^54–56^. The invoked single-photon superabsorption mechanism based on strong electronic correlations is consistent with recent reports of enhanced radiative rates^4,57–60^ and large multiexciton binding energies^51,52^ in CsPbBr_3_ perovskite QDs. Overall, our results match well with the original idea^2,5^ depicted in Fig. 1, which considers the absorption cross-section in large QDs as a collective response of *N* contributing unit cells that form a new giant dipole, with giant single-photon absorption and single-photon emission rates.

In conclusion, we demonstrate single-photon superabsorption at the bandgap (1S) transition in CsPbBr_3_ perovskite QDs at cryogenic temperatures, enabled by the material’s strong electronic correlations, a ‘textbook-like’ band structure, and synthetic access to size- and shape-pure QDs covering three orders of magnitude in volume. The experimentally established hallmarks of single-photon superabsorption, including the increase in bandgap absorption strength with increasing QD size, are quantitatively explained by CI-EMA calculations accounting for electronic correlations. Apparently, boosted absorption strength is yet another experimentally overlooked property of excitons in the weak-confinement regime, with exciton optical properties largely dominated by many-body correlations. The single-photon superabsorption discovered here in colloidal perovskite QDs could open new research avenues towards the development of more efficient lasers in the regimes of weak and strong light–matter coupling and aid the design of novel quantum light–matter interfaces in which a single superabsorbing QD is coupled to an optical mode of a high-quality-factor microcavity.

## Methods

### Materials

Lead bromide (PbBr_2_, 99.999%), caesium carbonate (99.9%), hexane (≥99%), oleic acid (OA, 90%), acetone (≥99.5%), phosphorus(v) oxychloride (99%), 2-aminoethan-1-ol (≥99.0%), acetic acid (>99.8%), triethylamine (99%) and 2-octyl-1-dodecanol (97%) were purchased from Sigma-Aldrich. *n*-octane (min. 99%) and lecithin (>97% from soy) were purchased from Carl Roth. Trioctylphosphine oxide (TOPO, min. 90%) was purchased from Strem Chemicals. Bis(2,4,4-trimethylpentyl)phosphinic acid (TMPPA, 90%) was purchased from Fluorochem. Mesitylene (99%) was purchased from Thermo Scientific Chemicals. ω-Hydroxyterminated polystyrenes (*M*_n_ = 900 P4465-SOH, *M*_n_ = 5,000 P18731-SOH) were purchased from Polymer Source.

### QD synthesis

Colloidal CsPbBr_3_ perovskite QDs were synthesized according to earlier work^42^^,^^61^ with slight modifications.

#### 0.04-M Pb stock solution for 3.5-nm and 5-nm CsPbBr_3_ QDs

The PbBr_2_ stock solution (0.04 M) was prepared by dissolving 2 mmol (0.734 g) of PbBr_2_ and 10 mmol (3.866 g) of TOPO into 10 ml of octane at 120 °C in a 40-ml vial. Once all the PbBr_2_ was dissolved (~30 min), the solution was cooled to room temperature and transferred to a big SCHOTT bottle and diluted by 40 ml of hexane. The stock solution was filtered after preparation over a 0.45-µl polytetrafluoroethylene (PTFE) filter and stored in air.

#### 0.04-M Pb stock solution for 7-nm CsPbBr_3_ QDs

The PbBr_2_ stock solution (0.04 M) was prepared by dissolving 1 mmol (0.367 g) of PbBr_2_ and 5 mmol (2.15 g) of TOPO into 5 ml of octane at 100 °C in a 20-ml vial. Once the entire PbBr_2_ was dissolved (~30 min), the solution was cooled to room temperature, transferred to a 40-ml vial and diluted with 20 ml of hexane. The stock solution was filtered after preparation over a 0.2-µl PTFE filter and stored in air.

#### 0.02-M Cs-TMPPA stock solution

The Cs-TMPPA stock solution (0.02 M) was prepared by loading 100 mg of caesium carbonate (0.614 mmol of Cs) together with 1 ml of TMPPA (3.154 mmol) and 2 ml of octane at 120 °C in a 40-ml vial. Once all the caesium carbonate was dissolved (~20 min), the stock solution was cooled to room temperature and 27 ml of hexane was added. The stock solution was filtered after preparation over a 0.2-µl PTFE filter and stored in air.

#### Lecithin stock solution (~0.13 M) for 3.6-nm and 5.2-nm CsPbBr_3_ QDs

This stock solution was prepared by dissolving 1 g of lecithin in 20 ml of hexane using an ultrasonic bath (30 min). After preparation, the stock solution was centrifuged, filtered over a 0.2-µl PTFE filter and stored in air.

#### Synthesis of 3.5-nm CsPbBr_3_ QDs

The filtered hexane (18 ml) was mixed with a PbBr_2_ stock solution (0.84 ml). Under heavy stirring, a 0.02-M Cs-TMPPA stock solution was injected (0.42 ml). After 1 min of QD growth, the lecithin solution was added to the crude CsPbBr_3_ solution (0.84 ml). After 50 s of growth, the solution was concentrated on a rotavap under a constant temperature. The QDs were precipitated from the concentrated solution (1.2 ml) by an excess of acetone (3.6 ml). The obtained pellet was dissolved in 1.2 ml of toluene and washed three times with a toluene/ethanol pair of solvent/antisolvent (1.2/1.8 ml, 1.2/1.5 ml and 1.2/1.2 ml at each washing step). The final washed pellet was redissolved in 0.4 ml of toluene.

#### Synthesis of 5-nm CsPbBr_3_ QDs

The filtered hexane (12 ml) was mixed with a PbBr_2_ stock solution (1.4 ml). Under heavy stirring, a 0.02-M Cs-TMPPA stock solution was injected (0.7 ml). After 1 min of QD growth, the lecithin solution was added to the crude CsPbBr_3_ solution (0.7 ml). After 13 min of growth, the solution was concentrated on a rotavap under constant temperature. The QDs were precipitated from the concentrated solution (3 ml) by an excess of acetone (9 ml). The obtained pellet was dissolved in 0.7 ml of toluene and washed three times with a toluene/ethanol pair of solvent/antisolvent (0.7/0.7 ml, 0.7/0.7 ml and 0.7/0.7 ml at each washing step). The final washed pellet was redissolved in 0.4 ml of toluene.

#### Synthesis of 7-nm CsPbBr_3_ QDs

The filtered hexane (4.5 ml) was mixed with a PbBr_2_ stock solution (2 ml). Under heavy stirring, a 0.02-M Cs-TMPPA stock solution was injected (1 ml). After 4 min of QD growth, the lecithin solution was added to the crude CsPbBr_3_ solution (1 ml). In 2 min after the addition of ligands, the crude solution was concentrated by evaporating hexane on a rotary evaporator down to less than 1 ml of residual solvent. To purify the QDs, ethyl acetate and acetone mixture (1:1) was used (crude solution:non-solvent, 1:3 v/v), followed by solubilization of the obtained NCs in 1 ml of anhydrous toluene. The purification procedure was repeated once more.

#### Synthesis of 17-nm and 30-nm CsPbBr_3_ QDs

The QDs were synthesized by adapting the PbBr_2_-TOPO approach from another work^42^, with a slow injection of Cs-OA and PbBr_2_-TOPO precursors at higher temperature. The QDs were capped with 2-ammonioethyl ω-poly(styrene)-yl phosphates (0.9k-PS-PEA and 5k-PS-PEA), precipitated twice with hexane, dispersed in toluene and further treated with 2-ammonioethyl 2-octyl-1-dodecyl phosphate. The phosphoethanolamine-based ligands were synthesized following an earlier protocol^43^.

### Thin-film sample processing

CsPbBr_3_ QD thin films on quartz substrates have been obtained by either spin coating or drop casting inside a N_2_-filled glovebox.

### Transmission electron microscopy

High-angle annular dark-field scanning transmission electron microscopy images were obtained using an FEI Titan Themis aberration-corrected microscope operated at 300 kV.

### Optical absorption measurements

Temperature-dependent optical absorption spectra have been acquired in the transmission geometry for CsPbBr_3_ QD thin films in a vacuum and mounted inside a liquid-helium cryostat (ARS DE204AE). Light from a white-light source (Ocean Insight DH2000BAL) was guided to the sample via a multimode fibre (Ocean Optics QP600-1-SR-BX), a collimator (Thorlabs CVH100-COL and LA4647) and a diaphragm to restrict the probe spot size to ~2 mm. The light transmitted through the sample was collected with a fibre coupler (Thorlabs CVH100-COL and LA4647) and guided to a broadband detector (Ocean Insight QE Pro spectrometer) through a round–linear fibre bundle (Thorlabs BFL200HS02). Energy-dependent absorbance spectra *A*(*E*) were obtained from the transmitted light intensity according to *A*(*E*) = −log_10_[(*I*_sample_(*E*) − *I*_dark_)/(*I*_substrate_(*E*) − *I*_dark_)], where *I*_sample_(*E*) and *I*_substrate_(*E*) are the recorded intensities after passing through the sample and substrate only, respectively, and *I*_dark_ is the spectrally constant dark counts.

The observation of negligible enhancement in the higher-energy transitions in the CI-EMA calculation even on the inclusion of electron–hole interaction and the similarity of low- and room-temperature absorption spectra at energies of at least 0.3 eV above the 1S transition suggests that the absorbance ratio of the 1S transition to such higher-energy transitions could, in principle, act as a proxy for the correlation-induced 1S enhancement. However, experimental inaccuracies may arise due to light scattering and poorly resolved spectral features arising from a spectral broadening exceeding the peak separations, both especially pronounced towards high energies. To address these experimental challenges, especially for ensembles of large QDs, we instead opt for the 1S–1P absorbance ratio *A*_1S_/*A*_1P_ as an experimentally more accessible but also more conservative estimate for the magnitude of the 1S enhancement. Both 1S and 1P peaks can be fitted with high accuracy for all thin films across our broad QD size range (Supplementary Fig. 3). Furthermore, we confirmed that reflection and scattering were negligible in the energy range around the 1S and 1P transitions and could be accounted for by a small energy-independent background.

To quantify the relative contributions by the 1S and collective 1P transitions, the obtained absorbance spectra were fitted in the near-gap region by a sum of three to four Gaussians, accounting for contributions from the 1S, collective 1P and collective 1D transitions (Supplementary Fig. 3). The relative absorbance strength *A*_1S_/*A*_1P_ of the 1S and collective 1P transition as reported in the main text is obtained from the ratio of the areas under the respective Gaussian peaks. This procedure accounts for the well-known observation that the respective absorbance peaks undergo thermally activated broadening arising from a phonon-induced redistribution of the oscillator strength.

### Configuration-interaction calculations

The used method for assessing the electronic structure of CsPbBr_3_ QDs of varying sizes is detailed in Supplementary Note 1, including details on the electron–hole pair correlations, one-photon absorption cross-section, temperature and line-broadening effects, as well as materials parameters and computational details.

## Online content

Any methods, additional references, Nature Portfolio reporting summaries, source data, extended data, supplementary information, acknowledgements, peer review information; details of author contributions and competing interests; and statements of data and code availability are available at 10.1038/s41566-025-01684-3.

## Supplementary information

Supplementary Information Supplementary Figs. 1–6, Notes 1–5 and Equations (1)–(20).

## Data Availability

The datasets generated and/or analysed during this study are available via the ETH Zürich Library at 10.3929/ethz-b-000728308.
