# Supplementary material for: Single-photon superabsorption in CsPbBr3 perovskite quantum dots
Source: Nat Photonics. 2025 May 21;19(8):864–70. doi: 10.1038/s41566-025-01684-3 (PMC12353793; doi:10.1038/s41566-025-01684-3)
Supplement: Supplementary file 1 — Supplementary Figs. 1–6, Notes 1–5 and Equations (1)–(20). [file 41566_2025_1684_MOESM1_ESM.pdf]

nature photonics

Supplementary information https://doi.org/10.1038/s41566-025-01684-3

Single-photon superabsorption in $CsPbBr_3$ perovskite quantum dots

In the format provided by the authors and unedited

Table of Contents

1. Supplementary Note 1. Theoretical model. 2
   1.1. Electronic calculations for a correlated electron-hole pair 2
   1.2. One-photon absorption cross-section 3
   1.3. Temperature effects and broadening of the line shape 4
   1.4. Material parameters and computational details 4
   1.5. Additional calculated optical absorption spectra 5
2. Supplementary Note 2. Absolute absorbance enhancement with QD size 7
3. Supplementary Note 3. Fitting of the experimental absorbance spectra 8
4. Supplementary Note 4. Connection of single-photon superabsorption to single-photon superradiance in individual $CsPbBr_3$ QDs 9
5. Supplementary Note 5. Coherence of the excitation field 11
6. References 12

1. Supplementary Note 1. Theoretical model.

1.1. Electronic calculations for a correlated electron-hole pair

The theoretical model considers a system of N_e quasi-particles in conduction states and N_h quasi-particle in the valence states confined in an external potential V_{ext} defined by the shape of the nanoparticle of interest. The total Hamiltonian reads

$$H_{eff} = \sum_A H_{kin}^A + V_{ext} + \sum_{AB} g_{AB}. \quad (1)$$

The kinetic part H_{kin}^A of quasi-particle A is described using the effective-mass approximation (EMA) as

$$H_{kin}^A = \frac{1}{2m^*}\nabla^2, \quad (2)$$

with m^* being the mass of the quasi-particle, which is either m_e or m_h for the electron and hole, respectively. For the QD shape and its confinement potential, we adopt cylindrical symmetry and denote L_z the length of the nanostructure and R_{eff} its radius. This ensures generalization to anisotropic QDs shapes in forthcoming publications. The confinement potential reads

$$V_{ext} = \begin{cases} 0, if\ |z| < \frac{L_z}{2}, r < R_{eff} \\ \propto, \text{otherwise.} \end{cases} \quad (3)$$

The solutions of the noninteracting part ($\sum_A H_{kin}^A + V_{ext}$) of the Hamiltonian provide noninteracting envelope wavefunctions,

$$\psi_{\nu n,n_z}(\vec{r}) = \frac{1}{\sqrt{2\pi}} e^{i\nu\phi} J_{\nu n}(r)\, \varsigma_{n_z}(z), \quad (4)$$

and noninteracting eigenvalues, *i.e.* confinement energies,

$$E_{\nu n,n_z}^0 = \frac{\hbar^2}{2m^*}\frac{1}{L_z^2}(\beta^2\alpha_{\nu n}^2 + \pi^2 n_z^2). \quad (5)$$

$J_{\nu n}(r)$ is the Bessel function of order $\nu = 0, \pm1, \pm2, \cdots$ and $\alpha_{\nu n}$ its nth root. The z-part $\varsigma_{n_z}(z)$ of the non-interacting wavefunction is given by

$$\varsigma_{n_z}(z) = \sqrt{\frac{2}{L_z}} \begin{cases} \cos\left(n_z \frac{\pi}{L_z} z\right), \text{if } n_z \text{ is odd}, \\ \sin\left(n_z \frac{\pi}{L_z} z\right), \text{if } n_z \text{ is even.} \end{cases} \quad (6)$$

Here, we restrict our analyses to isotropic QDs and choose

$$R_{eff} = \frac{L_z \alpha_{01}}{\sqrt{2}\pi}, \quad (7)$$

which ensures that the uncorrelated confinement energies are equal along the three directions.

We further proceed considering the Coulomb interaction between quasi-particles A and B,

$$g_{AB} = q_A q_B \frac{1}{\varepsilon_{eff}} \frac{1}{|\vec{r}_A - \vec{r}_B|}, \quad (8)$$

with $q_A = 1$ (-1) if A is a hole (electron) and ε_{eff} is the effective dielectric constant of the material. We treat electronic correlation at the level of the configuration-interaction (CI) framework. The solutions of

the noninteracting Hamiltonian (4-6) form a good basis set to implement the CI method. To simplify the notation, let us denote i the noninteracting quantum numbers $(\nu_i\, n_i, n_{z,i})$ of the ith single particle electron state $\psi_i^e(\vec{r})$. Similarly, we introduce j for the jth single particle hole state ($\psi_j^h(\vec{r})$). The tensor product $|\psi_i^e(\vec{r})\rangle \otimes |\psi_j^h(\vec{r})\rangle$ forms a single-exciton basis set. By evaluating the effective Hamiltonian (1) in this basis, we obtain the following CI matrix

$$H_{eff_{i'j',ij}} = (E_i^0 + E_j^0)\delta_{i',i}\delta_{j',j} + V_{i'j',ij}. \tag{9}$$

The Coulomb matrix elements read

$$V_{i'j',ij} = \langle i'j'|g_{AB}|ij\rangle, \tag{10}$$

with the following selection rules

$$\begin{cases} \nu_{i'} + \nu_{j'} = \nu_i + \nu_j \\ \Lambda_{i'} + \Lambda_{j'} = \Lambda_i + \Lambda_j \end{cases}. \tag{11}$$

Here, the parity quantum number Λ_i is defined as $mod(n_{z,i} + 1,2)$, which means that the z-component $\varsigma_{n_z}(z)$ has even (odd) parity if $n_{z,i}$ is odd (even). In other words, the total in-plane angular momentum ν and z-parity Λ are the conserved quantities for a cylindrical QD. In solving the eigenvalue problem defined in eq. (9), we obtain the eigensolutions

$$|\alpha\rangle = \sum_{ij} A_{ij}^{\alpha}\, |\psi_i^e(\vec{r})\rangle \otimes |\psi_j^h(\vec{r})\rangle \tag{12}$$

and

$$\sum_{ij} H_{eff_{i'j',ij}} A_{ij}^{\alpha} = \omega_\alpha A_{i'j'}^{\alpha}. \tag{13}$$

For each $|\alpha\rangle$, the eigensolutions of (12) are normalized

$$\sum_{ij} |A_{ij}^{\alpha}| = 1. \tag{14}$$

1.2. One-photon absorption cross-section

The one-photon absorption cross section at frequency ω reads[1,2]

$$\sigma^{(1)}(\omega) = \sum_\alpha F_\alpha^{(1)}(\omega_\alpha) g_\alpha(\omega - \omega_\alpha), \tag{15}$$

where $g_\alpha(\omega - \omega_\alpha)$ accounts for the broadening line shape for each of the transition centered at ω_α and $F_\alpha^{(1)}(\omega_\alpha)$ is the transition strength that is linked to the optical matrix element M_α

$$F_\alpha^{(1)}(\omega_\alpha) = \frac{4\pi^2}{3} \frac{{f_\varepsilon}^2}{\sqrt{\varepsilon_{out}}} \frac{1}{c_0 \omega_\alpha} |M_\alpha|^2. \tag{16}$$

Here, ε_{out} is the dielectric constant of the medium surrounding the QDs and f_ε the dielectric screening factor which is considered within the spherical approximation

$$f_\varepsilon = \frac{3\varepsilon_{out}}{2\varepsilon_{out}+\varepsilon_{opt}}, \quad (18)$$

with ε_{opt} the dielectric constant of the medium at optical frequencies. The optical matrix element reads

$$|M_\alpha|^2 = \left|\sum_{ij} A_{ij}^{\alpha} \langle \mathrm{T}\psi_j^h | \psi_i^e \rangle \langle J_j \| p \| J_i \rangle\right|^2 = \chi_{corr,\alpha}\, E_P. \quad (17)$$

Here, T is the time-reversal operator and E_P the Kane energy. $\chi_{corr,\alpha}$ denotes the correlation enhancement factor for exciton state $|\alpha\rangle$ and is given by

$$\chi_{corr,\alpha} = \left|\sum_{ij} A_{ij}^{\alpha}\, \delta_{-\nu_j,\nu_i}\, \delta_{n_j,n_i}\, \delta_{n_{z,j},n_{z,i}}\right|^2. \quad (19)$$

For the case of an uncorrelated electron-hole pair, in which the wavefunction is simply the product of single particle wavefunctions, $\chi_{corr,\alpha} = 1$.

1.3. Temperature effects and broadening of the line shape

The temperature dependence of the band gap is approximated by[3]

$$E_g(T) = E_g(0) + aT + b\left(e^{\frac{\hbar\omega_{LO}}{k_B T}} - 1\right)^{-1}. \quad (20)$$

In this expression, a describes the linear growth due to thermal expansion and electron-acoustic phonon coupling. The third term approximates the effect related to electron-LO phonon coupling.

In eq. (15), the function $g_\alpha(\omega - \omega_\alpha)$ aims at modeling the line broadening of each transition at energy ω_α. There are at least two sources of broadening: i) inhomogeneous broadening due to QD size dispersion, with a Gaussian standard deviation extracted from the CI calculations, and ii) predominantly homogeneous broadening due to temperature-dependent electron-phonon coupling. A detailed description as well as corresponding materials parameters can be found in the Supplementary Information of ref.3. Here, to emulate the experiment, we choose a size dispersion of 10%.

1.4. Material parameters and computational details

For the effective Hamiltonian derived within EMA, we took the same material parameters for $CsPbBr_3$ as those taken by Blundell et al.,[4] except for the external polystyrene matrix for which $\epsilon_{out} = 2.56$. With these parameters, the exciton Bohr radius $a_B = 3.07$ nm and the computed exciton binding energy for an isotropic QD (eq. 7) with $L_z = 10\, a_B$ amounts to 27.5 meV, in good agreement with the theoretical description based on Hartree-Fock orbitals[4] and the experimental exciton binding energy reported for bulk $CsPbBr_3$.[5]

The parameters used to model the temperature dependence of the band gap, eq. (20), are $a = 0.339$ meV/K,[6] $\hbar\omega_{LO} = 16$ meV,[7] and $b = 30$ meV. The latter is chosen to best reproduce the band gap at 270 K of ref. 5.

The wavefunction given by eq. (12) is expended in a basis set consisting of at least 12500 states. Each Coulomb matrix element, eq. (10), is evaluated up to a precision of the 7th decimal digit. To obtain the m lowest eigenvalues of the CI matrix (13), exact diagonalization is performed using the '*eigs*' built-in function of GNU Octave. Absorption spectra are computed considering the first hundred transitions, i.e., $m = 100$.

For the temperature-dependent absorption spectra displayed in Figs. S1c and S1d, the temperature step is 14.75 K.

1.5. Additional calculated optical absorption spectra

Fig. S1 shows that the computed optical absorption spectra at low temperature (15 K) exhibit distinctive absorption features, especially for the two first excitations. For a strongly confined QD with a size of $L_z = 5$ nm (see Fig. S1a), the QD size is close to the exciton Bohr radius and the wavefunction of the lowest excitation consists to 98% of the contribution from the pair $|\psi_i^e(\vec{r})\rangle \otimes |\psi_j^h(\vec{r})\rangle$ with the electron quantum numbers $(\nu_i\, n_i, n_{z,i}) = (0,1,1)$. The corresponding hole quantum numbers are completely determined by the selection rule implicit in eq. (19). The second peak receives major contributions from the pair states with $(\nu_i\, n_i, n_{z,i}) = (0,1,2)$ (51% in norm) and $(\nu_i\, n_i, n_{z,i}) = (\pm,1,1)$ (41%). Therefore, we identify the first two peaks as the "1S" and "1P" transitions in the order of increasing energy. For a QD with a size $L_z = 16$ nm (Fig. S1b), *i.e.,* a QD in the intermediate-to-weak confinement limit, the 1S and 1P peak remain clearly visible at 15 K, even after considering the expected broadening due to phonons and size distribution described in Supplementary Notes 1.3 and 1.4. The lowest "1S"-like peak comprises 74% contribution by $(\nu_i\, n_i, n_{z,i}) = (0,1,1)$ and 10% by $(\nu_i\, n_i, n_{z,i}) = (\pm,1,1)$. The "1P"-like peak comprises two transitions, at 2.360 eV and 2.661 eV, respectively. The first transition receives 37% contribution from $(\nu_i\, n_i, n_{z,i}) = (0,1,2)$ ($1P_z$), 8% from $(\nu_i\, n_i, n_{z,i}) = (\pm1,1,2)$, but also 3% from $(\nu_i\, n_i, n_{z,i}) = (0,1,1)$. The second transition receives 38% from $(\nu_i\, n_i, n_{z,i}) = (\pm1,1,1)$ ($1P_{x/y}$), 4% from $(\nu_i\, n_i, n_{z,i}) = (0,1,2)$, and 3% of $(\nu_i\, n_i, n_{z,i}) = (\pm2,1,1)$. Figs. S1c and S1d illustrate the computed temperature dependence of the absorbance for the two different QD sizes. For the small 5 nm QD, "1S" and "1P" peaks show discernable patterns at all temperatures, from 5 K to 300 K, whereas for the larger 16 nm QD, significant phonon-induced broadening washes out the peaks near room temperature.

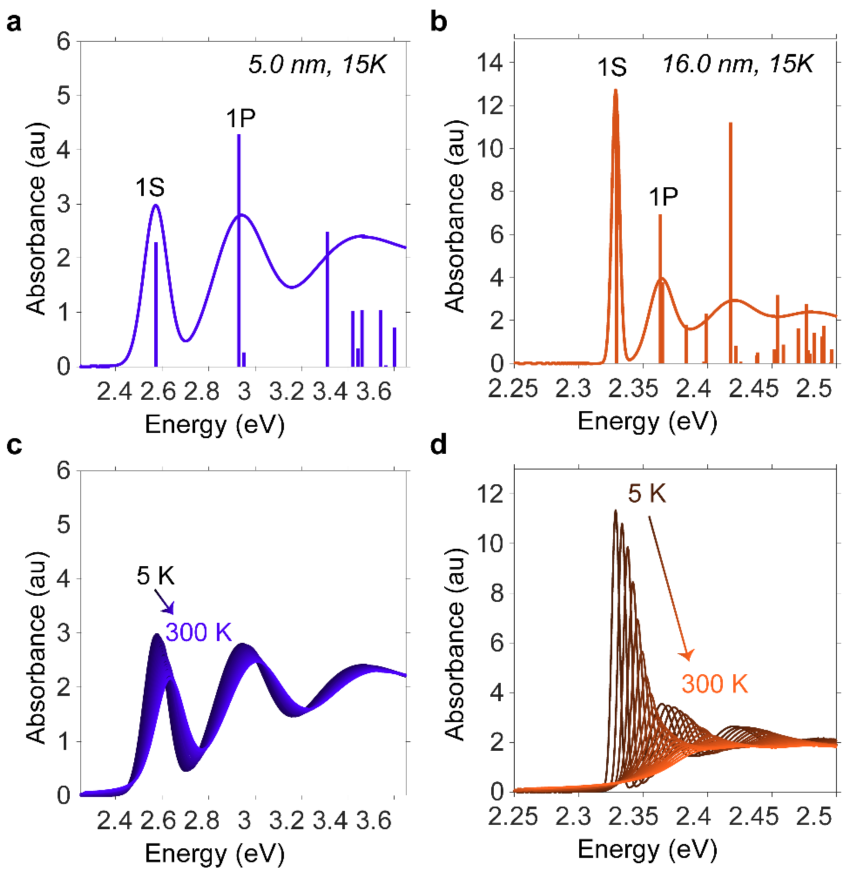

Figure S1 | Simulated absorbance versus excitation energy. Oscillator strength (bar) and absorbance (curve) computed at 15 K for a QD size of **(a)** $L_z = 5$ nm and **(b)** $L_z = 16$ nm. Absorbance computed for varying temperatures from 5 K to 300 K for **(c)** $L_z = 5$ nm and **(d)** $L_z = 16$ nm. The employed temperature step is 14.75 K and the arrows indicate increasing temperatures.

2. Supplementary Note 2. Absolute absorbance enhancement with QD size

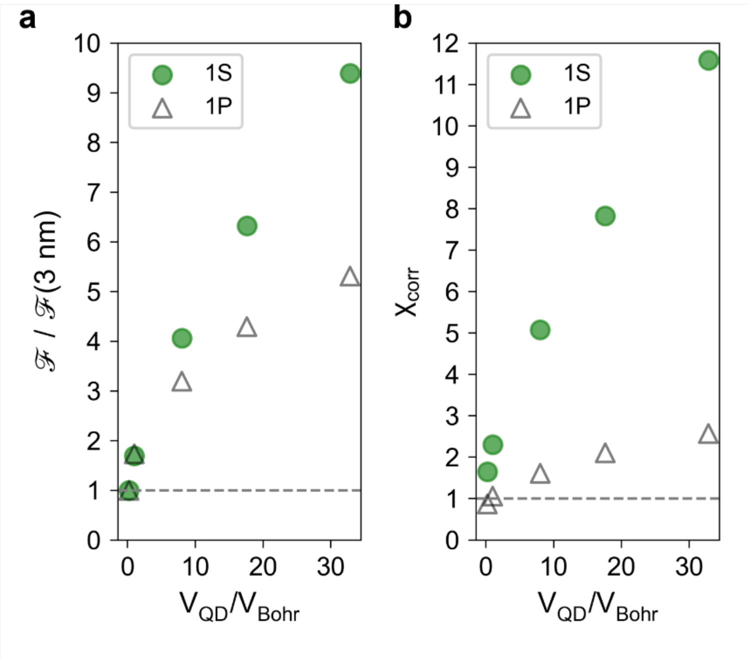

Figure S2 | Absolute correlation-induced absorbance enhancement with increasing QD size, calculated at 15 K and at the CI-EMA level of theory. (a) Transition strength ($\mathcal{F}$) of the 1S (green closed circles) and the 1P transition (grey open triangles), respectively, referenced to their values in the smallest 3 nm QD. **(b)** Correlation-induced enhancement (X_{corr}) of the 1S and 1P transition strength.

3. Supplementary Note 3. Fitting of the experimental absorbance spectra

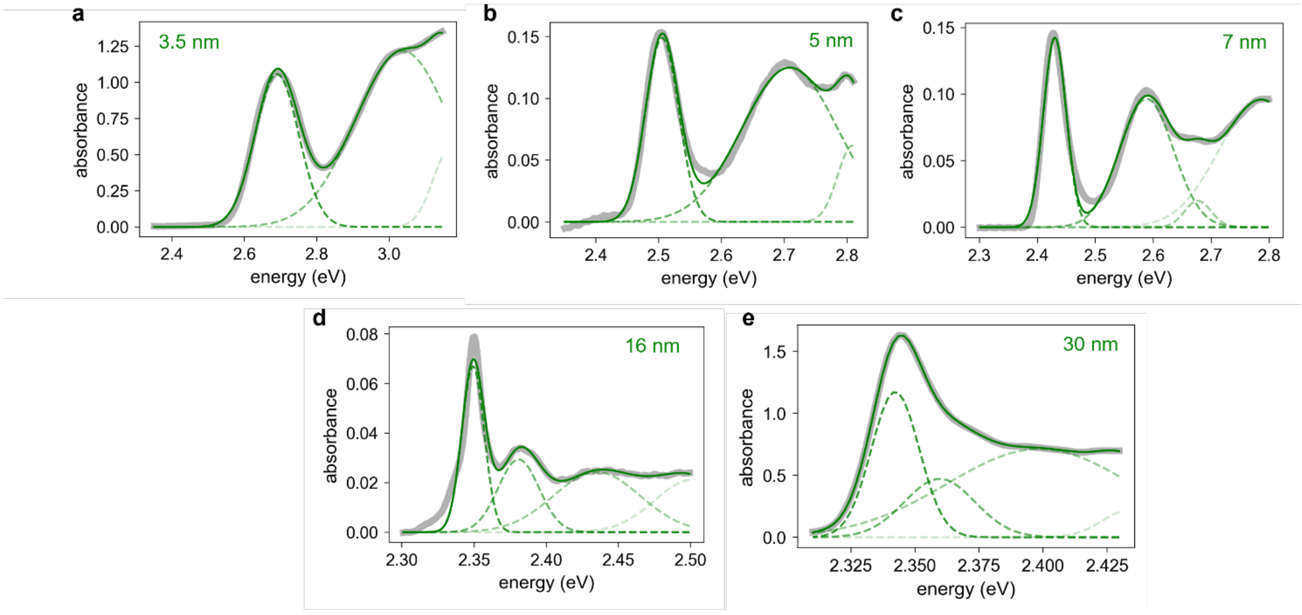

Figure S3 | Fitting of the $CsPbBr_3$ QD thin-film absorption spectra with a sum of Gaussians. (a) Experimental data (thick grey solid line), overall fit (green solid line), and individual fit contributions from the underlying excitonic transitions 1S, 1P, etc. (dashed green lines) for a thin film of 3.5 nm QDs. **(b)-(e)** Same for thin films comprising larger QDs with mean sizes of about 5 nm, 7 nm, 16 nm, and 30 nm, respectively. The respective fit contributions in (a)-(e) have been used to determine the size-dependent A_{1S}/A_{1P} absorbance ratio discussed in the main text.

4. Supplementary Note 4. Connection of single-photon superabsorption to single-photon superradiance in individual $CsPbBr_3$ QDs

Fig. S4 compares, for a large range of $CsPbBr_3$ QD sizes, the experimentally derived radiative rates from single-QD PL experiments, the experimentally derived 1S absorbance, and the calculated 1S transition strength. The obtained good agreement between the size-dependent trends of experimental data and calculation including e-h correlation suggests that the size-induced acceleration of the radiative decay and the enhancement of the absorption strength are connected and may both be traced back to the existence of strong e-h correlations enabling giant oscillator strength for very large perovskite QDs.

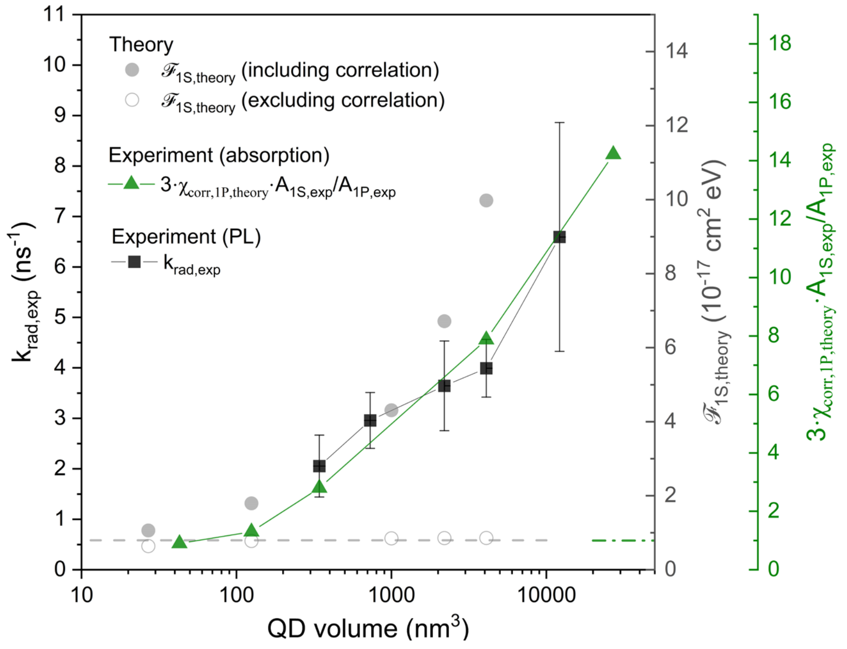

Figure S4 | Calculated QD-volume-dependent 1S transition strength and its relation to the measured 1S absorbance and radiative rate at cryogenic temperature. *Left axis*: experimental single-QD radiative rate $k_{rad,exp}$ at 4 K (black squares), presented as mean values +/- the standard deviation; the latter quantifies the sample-to-sample variations obtained from the measurement of typically 30 single QDs per QD size. *Right (grey) axis*: theoretical 1S transition strength $\mathcal{F}_{1S,theory}$ when excluding (open grey circles) or including e-h correlations (filled grey circles), calculated within the CI-EMA framework. *Second right (green) axis*: the experimental absorbance ratio at 15 K as reported in the main text ($A_{1S,exp}/A_{1P,exp}$) multiplied by the 1P/1S degeneracy ratio (3) and the calculated correlation-induced enhancement of the 1P oscillator strength ($\chi_{corr,1P,theory}$); this product (green triangles) serves as an experiment-derived and theory-aided estimate of the correlation-induced 1S absorbance enhancement with increasing QD volume, approaching unity (*i.e.*, no enhancement) for small and strongly confined QDs. All vertical axis limits have been chosen such that the strong-confinement limit of the experimental data matches the theoretical data without correlation: the limit of the left axis has been chosen such that the theoretical $\mathcal{F}_{1S,theory}$ without correlation (right axis) corresponds to a rate of about 1/2 ns^{-1} (grey dashed line), close to a recent estimate of 1/2.5 ns^{-1} by Blundell et al.;[4] equally, the limit of the second right axis has been chosen such that the

absence of 1S enhancement (corresponding to a value of unity; green dash-dotted line) matches the theoretical $\mathcal{F}_{1S,theory}$ without correlation.

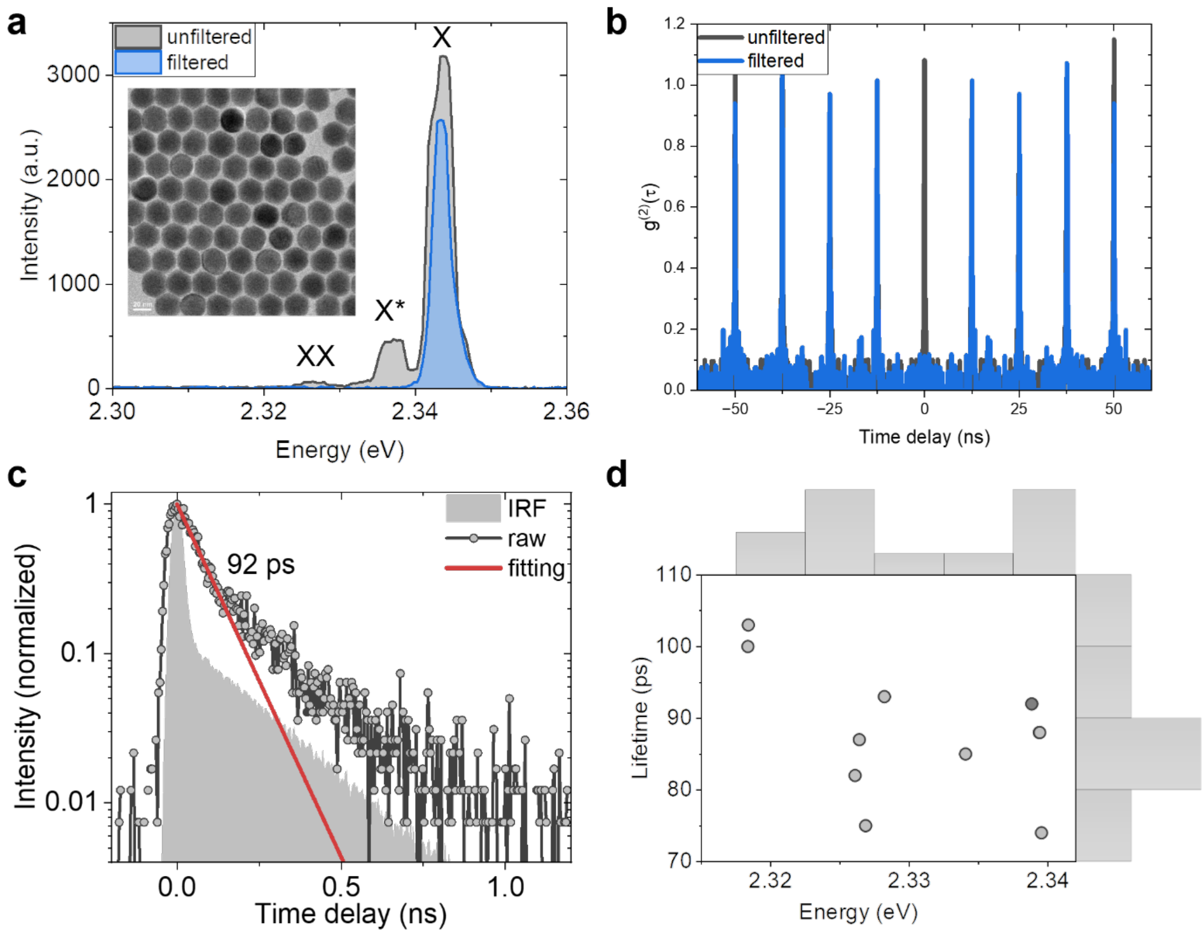

Figure S5 | Single-photon superradiance from excitons in a large individual $CsPbBr_3$ QD with a diameter of about 26.5 nm at 4 K. (a) Single-QD PL spectrum excited by pulsed excitation (480 nm; 80 MHz) with a fluence of 4.7 nJ/cm^2, featuring emission contributions from excitons (X), trions (X*), and biexcitons (XX), respectively (grey shaded area); spectral filtering with a short-pass filter suppresses the contributions from X* and XX (blue shaded area). **(b)** the second-order intensity correlation function $g^{(2)}(t)$ shows that the spectral filtering applied in (a) induces strong anti-bunching with $g^{(2)}(t=0)<0.1$, indicative of highly pure single-photon emission (blue trace). **(c)** Rapid radiative decay from a representative individual QD (black line with markers), characterized by a lifetime of 92 ps (obtained from single-exponential fitting; red line), close to the instrument-response function of the setup (IRF; grey shaded area). **(d)** statistics of the radiative lifetime for several individual $CsPbBr_3$ QDs from the same synthesis batch.

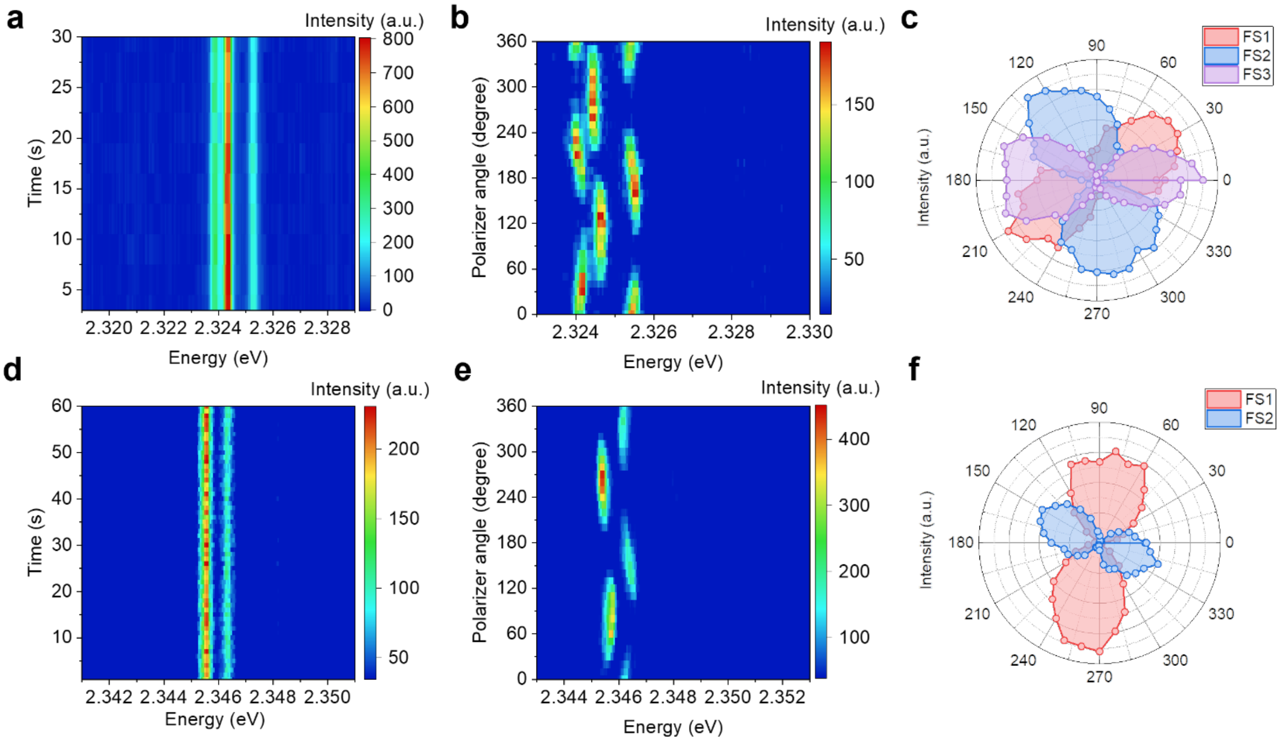

Figure S6 | Excitonic fine structure of large individual $CsPbBr_3$ QDs with a diameter of about 26.5 nm, featuring triplet emission ((a)-(c)) and doublet emission ((d)-(f)). (a) Temporal stability of the triplet emission. (b) Angle-resolved triplet emission spectrum, obtained by varying the angle of a linear polarizer in the detection path. (c) Angular pattern of the three fine structure lines (labelled FS1, FS2, and FS3, respectively), displaying linear polarization. (d)-(f) corresponding temporal stability, angle-resolved emission spectrum, and angular pattern for an individual QD featuring a doublet emission.

5. Supplementary Note 5. Coherence of the excitation field

We note that in the single-photon superabsorption mechanism discussed in the main text, there is no specific requirement for the coherence of the excitation light, since correlations among the photogenerated carriers are established almost instantaneously during the ground-to-excited state transition. In our implementation, we have used a *cw* broadband source (halogen lamp) whose generated light possesses very poor coherence.

6. References

1 Nguyen, T. P. T. *A theoretical study of correlation effects of N electrons in semiconductor nanocrystals : applications to optoelectronic properties of perovskite nanocrystals*, Nanyang Technological University, (2020).

2 Nguyen, T. P. T., Blundell, S. A. & Guet, C. One-photon absorption by inorganic perovskite nanocrystals: A theoretical study. *Physical Review B* **101** (2020). https://doi.org/10.1103/PhysRevB.101.195414

3 Akkerman, Q. A. *et al.* Controlling the nucleation and growth kinetics of lead halide perovskite quantum dots. *Science* **377**, 1406-1412 (2022). https://doi.org/10.1126/science.abq3616

4 Blundell, S. A. & Guet, C. All-order correlation of single excitons in nanocrystals using a k.p envelope-function approach: Application to lead halide perovskites. *Physical Review B* **105**, 155420 (2022). https://doi.org/10.1103/PhysRevB.105.155420

5 Yang, Z. *et al.* Impact of the Halide Cage on the Electronic Properties of Fully Inorganic Cesium Lead Halide Perovskites. *ACS Energy Letters* **2**, 1621-1627 (2017). https://doi.org/10.1021/acsenergylett.7b00416

6 Shibata, K., Yan, J., Hazama, Y., Chen, S. & Akiyama, H. Exciton Localization and Enhancement of the Exciton–LO Phonon Interaction in a $CsPbBr_3$ Single Crystal. *The Journal of Physical Chemistry C* **124**, 18257-18263 (2020). https://doi.org/10.1021/acs.jpcc.0c06254

7 Ramade, J. *et al.* Exciton-phonon coupling in a $CsPbBr_3$ single nanocrystal. *Applied Physics Letters* **112** (2018). https://doi.org/10.1063/1.5018413
